# Prevalence and antibiotic resistance of pathogens isolated from neurosurgical patients with postoperative central nervous system infections in a tertiary hospital in North China

**DOI:** 10.3389/fpubh.2025.1601107

**Published:** 2025-06-10

**Authors:** Fan Yang, Jingnan Chen, Mengjie Zhao, Hejun Zhao, Bin Yang, Xuequan Feng

**Affiliations:** ^1^Department of Neurosurgery, Tianjin First Central Hospital, Nankai University, Tianjin, China; ^2^TEDA Institute of Biological Sciences and Biotechnology, Nankai University, Tianjin, China; ^3^Department of Endocrinology and Metabolism, Tianjin First Central Hospital, Nankai University, Tianjin, China

**Keywords:** neurosurgery, postoperative central nervous system infection, pathogens, prevalence, antibiotic resistance

## Abstract

**Background:**

Postoperative central nervous system infection (PCNSI) is a serious complication following neurosurgery. Effective clinical management of PCNSI requires prompt antibiotic administration based on the identification of the causative pathogens and antibiotic resistance. This study aimed to investigate the causative pathogens of PCNSI and their antibiotic resistance profiles, which could help clinicians initiate appropriate empirical antibiotic therapy.

**Methods:**

The distribution and antimicrobial resistance of pathogens in patients with PCNSI from January 2014 to December 2023 were analyzed retrospectively. Cerebrospinal fluid samples were aseptically collected and subjected to standard microbiological methods for bacterial isolation and identification. Antibiotic sensitivity testing was performed via the Kirby–Bauer disk diffusion agar method.

**Results:**

A total of 396 patients were diagnosed with PCNSI, and 385 pathogens were identified from these patients. The percentages of Gram-positive bacteria, Gram-negative bacteria, and fungi were 56.10, 41.30, and 2.60%, respectively. The predominant pathogens among the Gram-positive bacteria were coagulase-negative staphylococci (29.09%), whereas *Acinetobacter baumannii* (14.29%) was the most common Gram-negative bacterium. Compared with those from 2014 to 2018, the proportions of *Enterococcus* and *Acinetobacter baumannii* increased markedly from 2019 to 2023. Antimicrobial susceptibility testing revealed that all Gram-positive bacteria had 100% sensitivity to vancomycin and linezolid, whereas imipenem, meropenem, and amikacin were most effective against Gram-negative bacteria.

**Conclusion:**

Gram-positive bacteria, especially coagulase-negative staphylococci, were the predominant pathogens causing PCNSI. Furthermore, several Gram-negative species, especially *Klebsiella pneumoniae* and *Acinetobacter baumannii*, showed concerning trends of increasing resistance to common antibiotics. *Acinetobacter baumannii* showed an increasing proportion of infections, posing a clinical challenge due to the limited number of effective antibiotics.

## 1 Introduction

Neurosurgical procedures, such as craniotomy, cerebrospinal fluid shunting, and external ventricular drain insertion, may impair immune function in patients, leading to a high incidence of complications (1–3). Postoperative central nervous system infection (PCNSI) is one of the most serious complications after neurosurgical procedures, resulting in poor prognosis and substantial health care costs (4–6). Epidemiological data indicate that the prevalence of PCNSI following neurosurgery ranges between 0.7 and 8.9%, with a mortality rate of approximately 5.0% among affected patients (7, 8).

Given the serious implications of PCNSI after neurosurgical procedures, prompt treatment of PCNSI in infected neurosurgical patients with prophylactic and empirical antibiotics is essential. The selection of appropriate antibiotics should be based on a comprehensive understanding of pathogen prevalence patterns and emerging resistance profiles (9). However, the growing number of neurosurgical procedures in recent years has potentially altered the epidemiological characteristics and clinical spectrum of postneurosurgical meningitis, complicating the existing empirical antibiotic therapy for PCNSI (10). In addition, the widespread use of broad-spectrum empirical antibiotics has increased the emergence of antibiotic resistance, consequently diminishing the therapeutic efficacy of empirical treatment against common PCNSI pathogens (11). Therefore, in recent decades, an urgent need to understand the characteristics of pathogens that cause PCNSI and antimicrobial resistance has emerged, with the aim of providing the most up-to-date insight to guide the use of empirical antibiotics.

Currently, existing data regarding pathogen distribution patterns and antibiotic sensitivity are inadequate for developing strategies for PCNSI prevention, diagnosis, and therapeutic management. This retrospective study presents pathogen prevalence and resistance patterns in neurosurgical patients with PCNSI at a tertiary hospital in North China, aiming to obtain a better understanding of PCNSI after neurosurgical procedures in the past decade.

## 2 Materials and methods

### 2.1 Ethical aspects

The study protocol was approved by the Research Ethics Committee of Tianjin First Center Hospital. The Ethics Committee granted permission to access the raw data and approved the waiver of informed consent to participate in this study due to its retrospective design. All patient data were anonymized before the analysis.

### 2.2 Study subjects and data collection

This retrospective study was carried out at Tianjin First Center Hospital, which is one of the largest comprehensive medical centers in northern China. This institution is equipped with 50 specialty departments and 3,200 inpatient beds, serving an annual patient volume of over 2 million. From January 2014 to December 2023, a total of 6,912 patients underwent neurosurgical procedures in the Department of Neurosurgery. Patients were administered standardized perioperative antibiotic prophylaxis (1–2 g of cefazolin or 0.6 g of clindamycin for cephalosporin allergy), followed by a 24-h postoperative course. Strict sterile techniques were implemented in accordance with the WHO surgical safety standards, specifically including preoperative hair removal for cranial surgery; sequential skin antisepsis using betadine and alcohol scrub; and sterile field establishment with antimicrobial drapes.

A total of 396 patients diagnosed with PCNSI and admitted to the Department of Neurosurgery from January 2014 to December 2023 were included. To identify meaningful long-term epidemiological trends while minimizing the impact of transient fluctuations, patients were divided into two groups based on the date of presentation: the first period was from January 2014 to December 2018, and the second period was from January 2019 to December 2023. Supplementary Figure S1 illustrates the flowchart of the study enrollment and research process.

### 2.3 Inclusion and exclusion criteria

The inclusion criteria for the study subjects were as follows: (i) patients were 18 years or older; (ii) patients had undergone elective or emergency neurosurgical procedures during the study period; (iii) patients who did not have infectious diseases before the neurosurgical procedures; and (iv) patients whose clinical records were complete, including age, sex, type of surgical procedure, and the operative duration.

The exclusion criteria for the study subjects were as follows: (i) patients presented with severe organ dysfunction; (ii) patients whose initial neurosurgical procedure was performed at another institution; and (iii) patients who had received preoperative antimicrobial treatment for other reasons, such as preexisting infections.

### 2.4 Diagnostic criteria

The diagnosis of PCNSI was established based on the criteria set by the Centers for Disease Control and Prevention (12), with minor adjustments. The inclusion criteria were as follows: (i) positive cerebrospinal fluid (CSF) culture after neurosurgery; (ii) typical clinical manifestations of PCNSI, such as fever (body temperature > 38°C), headache, stiff neck, meningeal signs, cranial nerve signs, altered level of consciousness, or mental confusion; (iii) an increase in white cells, elevated protein levels, and/or a decreased glucose concentration (CSF/serum glucose ratio < 0.3 or CSF glucose < 50 mg/dL in cases where serum glucose data are unavailable); and (iv) antibiotic treatment prescribed by the attending clinician.

### 2.5 Isolation of strains and identification methods

CSF samples were collected upon suspicion of infection by either lumbar puncture or percutaneous tapping of the fluid from the shunt reservoir as per standard procedures. The CSF samples were cultured on blood agar, chocolate agar, and MacConkey agar plates, followed by incubation at 37°C for 48 h. CSF-negative cultures were excluded. All CSF-positive culture pathogens were identified to the species level using standard microbiological methods.

### 2.6 Antibiotic susceptibility testing

Antimicrobial susceptibility profiles were determined via the Kirby–Bauer disk diffusion method, with the results interpreted according to the Clinical and Laboratory Standards Institute guidelines. Three control American Type Culture Collection (ATCC) organisms, namely, *Escherichia coli* ATCC 25922, *Pseudomonas aeruginosa* ATCC 27853, and *Staphylococcus aureus* ATCC 25923, were used as quality control strains.

### 2.7 Statistical analysis

Statistical analysis was performed using SPSS (version 18.0; SPSS Inc., Chicago, IL, United States). The data are expressed as frequencies and percentages. Statistical tests for between-group comparisons of isolated pathogens and antibacterial susceptibility were performed using the *χ*^2^ and Fisher’s exact tests. Differences for which *p* < 0.05 were considered statistically significant.

## 3 Results

### 3.1 Distribution of pathogens causing PCNSI

Between January 2014 and December 2023, a total of 6,912 patients underwent neurosurgical procedures in the Department of Neurosurgery. Among these patients, 396 patients (5.73%) were diagnosed with PCNSI. The demographic and clinical characteristics of these patients with PCNSI are comprehensively presented in Supplementary Table S1. A comparative analysis was subsequently conducted, and the results indicated that there were no statistically significant differences in these clinical characteristics between the two study periods.

**Figure 1:**
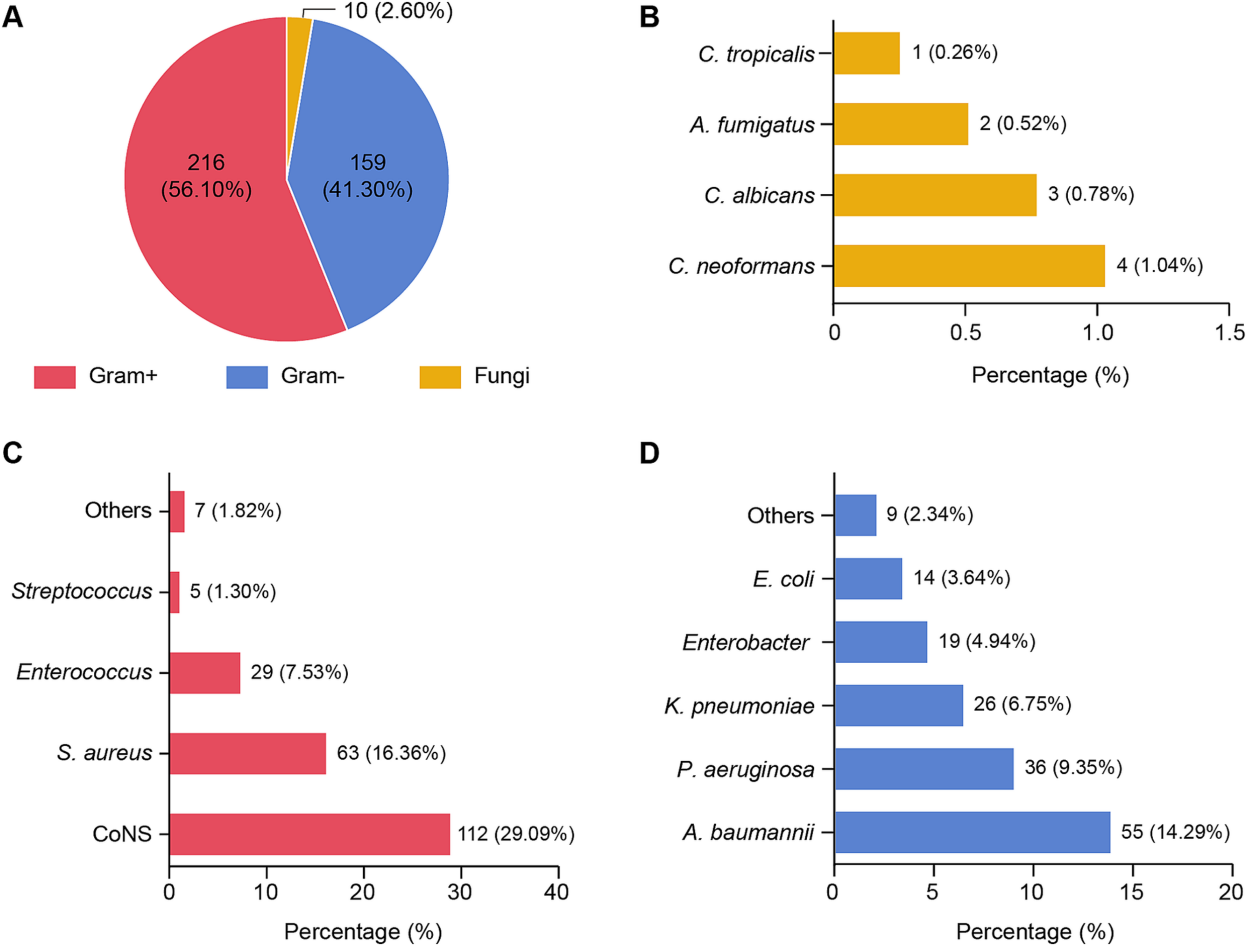
Distribution of pathogens isolated from patients with PCNSI 2014–2023. **(A)** Pie chart showing the distribution of 385 pathogens. Each pie slice represents a type of pathogen. **(B–D)** Bar graphs showing the proportions of different species of pathogens among fungi **(B)**, Gram-positive bacteria **(C)**, and Gram-negative bacteria **(D)**. The values represent the number and/or percentage of each pathogen. Gram+, Gram-positive bacteria; Gram−, Gram-negative bacteria; CoNS, coagulase-negative staphylococci; *Staphylococcus aureus*, *Staphylococcus aureus*; *Acinetobacter baumannii*, *Acinetobacter baumannii*; *Pseudomonas aeruginosa*, *Pseudomonas aeruginosa*; *Klebsiella pneumoniae*, *Klebsiella pneumoniae*; *Escherichia coli*, *Escherichia coli*; *C. neoformans*, *Cryptococcus neoformans*; *C. albicans*, *Candida albicans*; *A. fumigatus*, *Aspergillus fumigatus*; *C. tropicalis*, *Candida tropicalis*. The abbreviations used in this figure are the same as those used in Figures 2–5.

A total of 385 pathogens were identified from the 396 patients diagnosed with PCNSI. The pathogens responsible for PCNSI are presented in Table 1 and Figure 1. The predominant pathogens were Gram-positive bacteria (56.10%), whereas Gram-negative bacteria and fungi accounted for 41.30 and 2.60% of the pathogens, respectively (Table 1 and Figure 1A). Among the Gram-positive bacteria, the most common were coagulase-negative staphylococci, followed by *Staphylococcus aureus*, *Enterococcus*, and *Streptococcus* (Table 1 and Figure 1C). Among the Gram-negative pathogens, the most common were *Acinetobacter baumannii*, *Pseudomonas aeruginosa*, *Klebsiella pneumoniae*, *Enterobacter*, and *Escherichia coli* (Table 1 and Figure 1D). Among the fungi, *Cryptococcus neoformans*, *Candida albicans*, *Aspergillus fumigatus*, and *Candida tropicalis* accounted for 1.04, 0.78, 0.52, and 0.26% of the total pathogens, respectively (Table 1 and Figure 1B). Among all the isolated pathogens, the top five distributed pathogens were coagulase-negative *staphylococci*, *Staphylococcus aureus*, *Acinetobacter baumannii*, *Pseudomonas aeruginosa*, and *Enterococcus*, accounting for 29.09, 16.36, 14.29, 9.35, and 7.53% of the total pathogens, respectively.

**Table 1:** Pathogens isolated from patients with PCNSI from 2014 to 2023.

Pathogens	Frequency	Percentage (%)
Total	385	100.00
Gram-positive bacteria	216	56.10
Coagulase-negative staphylococci	112	29.09
*Staphylococcus aureus*	63	16.36
*Enterococcus*	29	7.53
*Streptococcus*	5	1.30
Others	7	1.82
Gram-negative bacteria	159	41.30
*Acinetobacter baumannii*	55	14.29
*Pseudomonas aeruginosa*	36	9.35
*Klebsiella pneumoniae*	26	6.75
*Enterobacter*	19	4.94
*Escherichia coli*	14	3.64
Others	9	2.34
Fungi	10	2.60
*Cryptococcus neoformans*	4	1.04
*Candida albicans*	3	0.78
*Aspergillus fumigatus*	2	0.52
*Candida tropicalis*	1	0.26

Among the 396 patients diagnosed with PCNSI in our study, 6 (1.52%) had polymicrobial infections, and a total of 13 pathogens from 7 species were identified. One of these patients was infected with more than two pathogen species. The causative pathogens isolated from the CSF samples with polymicrobial infections are listed in Supplementary Table S2.

### 3.2 Distribution of pathogens among different periods

**Figure 2:**
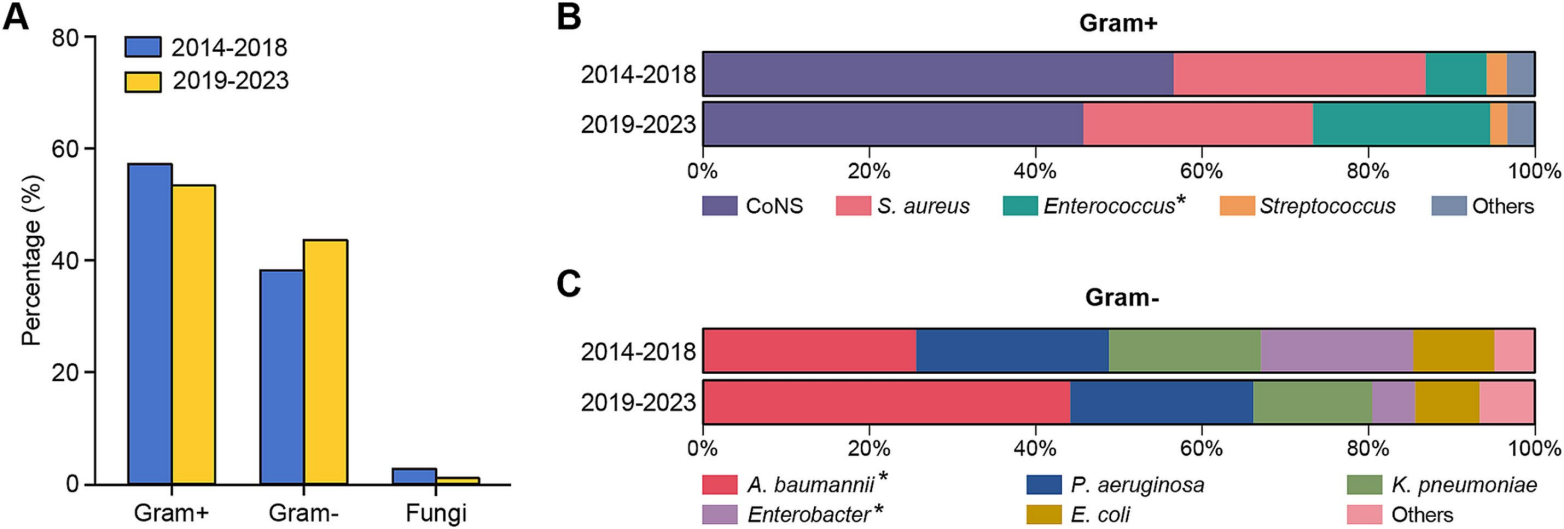
Distribution of different types of pathogens isolated from patients with PCNSI during different periods. **(A)** Bar graphs showing the percentages of Gram-positive bacteria, Gram-negative bacteria, and fungi causing PCNSI in different periods. **(B,C)** Bar diagram comparing the distributions of the predominant bacterial proportions among Gram-positive bacteria **(B)** and Gram-negative bacteria **(C)** isolated from patients with PCNSI in different periods. *The proportion of strains significantly differed between the two periods.

For comparative analysis, patients were divided into two groups based on the date of presentation: the first period was from January 2014 to December 2018, and the second period was from January 2019 to December 2023. The variations in pathogen distribution across different periods are shown in Table 2 and Figure 2.

**Table 2:** Comparison of the distribution of pathogens isolated from patients with PCNSI in different periods.

Gram-positive bacteria	2014–2018 (*n* = 122)		2019–2023 (*n* = 94)	
	Frequency	Percentage (%)	Frequency	Percentage (%)
Total	122	100.00	94	100.00
Coagulase-negative staphylococci	69	56.56	43	45.74
*Staphylococcus aureus*	37	30.33	26	27.66
*Enterococcus* ^a^	9	7.38	20	21.28
*Streptococcus*	3	2.46	2	2.13
Others	4	3.28	3	3.19

Gram-negative bacteria	2014–2018 (*n* = 82)		2019–2023 (*n* = 77)	
	Frequency	Percentage (%)	Frequency	Percentage (%)
Total	82	100.00	77	100.00
*Acinetobacter baumannii* ^b^	21	25.61	34	44.16
*Pseudomonas aeruginosa*	19	23.17	17	22.08
*Klebsiella pneumoniae*	15	18.29	11	14.29
*Enterobacter* ^c^	15	18.29	4	5.19
*Escherichia coli*	8	9.76	6	7.79
Others	4	4.88	5	6.49

Fungi	2014–2018 (*n* = 7)		2019–2023 (*n* = 3)	
	Frequency	Percentage (%)	Frequency	Percentage (%)
Total	7	100.00	3	100.00
*Cryptococcus neoformans*	3	42.86	1	33.33
*Candida albicans*	2	28.57	1	33.33
*Aspergillus fumigatus*	2	28.57	0	0.00
*Candida tropicalis*	0	0.00	1	33.33

^a^*χ*^2^ test, *p* = 0.003; OR = 0.2974; 95% CI: 0.1218–0.6834.

^b^*χ*^2^ test, *p* = 0.0140; OR = 0.4354; 95% CI: 0.2225–0.8597.

^c^Fisher’s exact test, *p* = 0.0137, OR = 4.086, 95% CI: 1.339–11.69.

Gram-positive bacteria were consistently the dominant pathogens causing PCNSI in the neurosurgical patients for the 10 years of the study period (Figure 2). The percentages of Gram-positive bacteria among all CSF-positive culture isolates in the first and second halves of the overall study period were 57.82 and 54.02%, respectively (Figure 2A). The proportion of *Enterococcus* bacteria among the Gram-positive bacteria increased significantly from 7.38% in the period from 2014 to 2018 to 21.28% in the period from 2019 to 2023 (Table 2 and Figure 2B). In terms of the distribution of Gram-negative bacteria, the proportion of *Acinetobacter baumannii* increased significantly from 25.61% in the first period to 44.16% in the second period, whereas that of *Enterobacter* decreased from 18.29 to 5.19% (Table 2 and Figure 2C). The isolation rates of the other strains did not significantly differ during the different periods.

### 3.3 Antimicrobial susceptibility of Gram-positive bacteria

**Figure 3:**
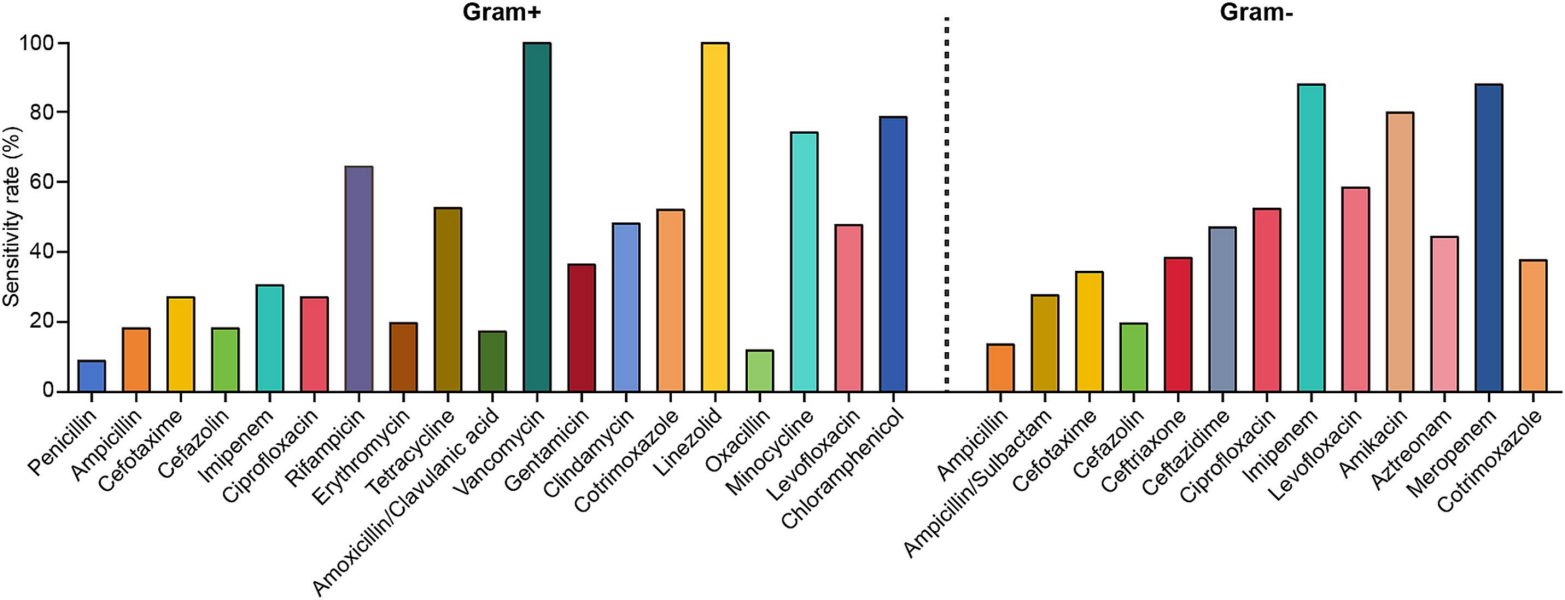
Antimicrobial susceptibility of the predominant Gram-positive and Gram-negative bacteria causing PCNSI from 2014 to 2023.

The antibiotic sensitivities of the predominant Gram-positive bacteria causing PCNSI from 2014 to 2023 are shown in Table 3 and Figure 3. Antimicrobial susceptibility analysis revealed that all the detected Gram-positive bacteria were 100% susceptible to vancomycin and linezolid. In addition, coagulase-negative staphylococci were highly susceptible to minocycline, rifampicin, and tetracycline. The drug susceptibility profile of *Staphylococcus aureus* revealed greater susceptibility to chloramphenicol, cotrimoxazole, and clindamycin but resistance to penicillin. *Enterococcus* strains were resistant to oxacillin, cefazolin, cotrimoxazole, and cefotaxime, whereas they presented relatively high sensitivity to imipenem, chloramphenicol, and ampicillin.

**Table 3:** Antimicrobial susceptibility of the predominant Gram-positive bacteria causing PCNSI from 2014 to 2023.

Antibiotic	Gram-positive bacteria (*n* = 204)		Coagulase-negative staphylococci (*n* = 112)		*Staphylococcus aureus* (*n* = 63)		*Enterococcus* (*n* = 29)	
	Frequency	Sensitivity rate (%)	Frequency	Sensitivity rate (%)	Frequency	Sensitivity rate (%)	Frequency	Sensitivity rate (%)
Penicillin	19	9.31	8	7.14	0	0.00	11	37.93
Ampicillin	38	18.63	14	12.50	6	9.52	18	62.07
Cefotaxime	56	27.45	27	24.11	29	46.03	0	0.00
Cefazolin	38	18.63	20	17.86	18	28.57	0	0.00
Imipenem	63	30.88	35	31.25	9	14.29	19	65.52
Ciprofloxacin	56	27.45	34	30.36	15	23.81	7	24.14
Rifampicin	132	64.71	99	88.39	27	42.86	6	20.69
Erythromycin	41	20.10	29	25.89	10	15.87	2	6.90
Tetracycline	108	52.94	93	83.04	10	15.87	5	17.24
Amoxicillin/Clavulanic acid	36	17.65	23	20.54	9	14.29	4	13.79
Vancomycin	204	100.00	112	100.00	63	100.00	29	100.00
Gentamicin	75	36.76	58	51.79	11	17.46	6	20.69
Clindamycin	99	48.53	34	30.36	57	90.48	8	27.59
Cotrimoxazole	107	52.45	49	43.75	58	92.06	0	0.00
Linezolid	204	100.00	112	100.00	63	100.00	29	100.00
Oxacillin	25	12.25	15	13.39	10	15.87	0	0.00
Minocycline	152	74.51	108	96.43	30	47.62	14	48.28
Levofloxacin	98	48.04	76	67.86	13	20.63	9	31.03
Chloramphenicol	161	78.92	81	72.32	61	96.83	19	65.52

### 3.4 Antimicrobial susceptibility of Gram-negative bacteria

The antibiotic sensitivities of the predominant Gram-negative bacteria causing PCNSI from 2014 to 2023 are shown in Table 4 and Figure 3. The results revealed that the Gram-negative bacteria detected in this study presented high levels of susceptibility to imipenem, meropenem, and amikacin. In addition, more than 60% of *Acinetobacter baumannii* strains were sensitive to levofloxacin, whereas they displayed low sensitivity (<50%) to other antimicrobial drugs. *Pseudomonas aeruginosa* was highly sensitive to levofloxacin but resistant to ampicillin/sulbactam and cotrimoxazole. *Klebsiella pneumoniae*, *Enterobacter*, and *Escherichia coli* exhibited 100% susceptibility to imipenem and meropenem, and they were all highly resistant to ampicillin (sensitivity rate <11%). However, levofloxacin sensitivity was much greater for *Klebsiella pneumoniae* than for *Enterobacter* and *Escherichia coli*, whereas the sensitivity of *Enterobacter* to ampicillin/sulbactam was much lower than that of *Klebsiella pneumoniae* and *Escherichia coli*.

**Table 4:** Antimicrobial susceptibility of the predominant Gram-negative bacteria causing PCNSI from 2014 to 2023.

Antibiotic	Gram-negative bacteria (*n* = 150)		*Acinetobacter baumannii* (*n* = 55)		*Pseudomonas aeruginosa* (*n* = 36)		*Klebsiella pneumoniae* (*n* = 26)		*Enterobacter* (*n* = 19)		*Escherichia coli* (*n* = 14)	
	Frequency	Sensitivity rate (%)	Frequency	Sensitivity rate (%)	Frequency	Sensitivity rate (%)	Frequency	Sensitivity rate (%)	Frequency	Sensitivity rate (%)	Frequency	Sensitivity rate (%)
Ampicillin	21	14.00	10	18.18	7	19.44	1	3.85	2	10.53	1	7.14
Ampicillin/Sulbactam	42	28.00	16	29.09	0	0.00	15	57.69	1	5.26	10	71.43
Cefotaxime	52	34.67	18	32.73	5	13.89	11	42.31	10	52.63	8	57.14
Cefazolin	30	20.00	3	5.45	5	13.89	13	50.00	2	10.53	7	50.00
Ceftriaxone	58	38.67	24	43.64	7	19.44	13	50.00	7	36.84	7	50.00
Ceftazidime	71	47.33	20	36.36	20	55.56	18	69.23	6	31.58	7	50.00
Ciprofloxacin	79	52.67	26	47.27	22	61.11	15	57.69	13	68.42	3	21.43
Imipenem	132	88.00	44	80.00	29	80.56	26	100.00	19	100.00	14	100.00
Levofloxacin	88	58.67	34	61.82	31	86.11	19	73.08	2	10.53	2	14.29
Amikacin	120	80.00	41	74.55	28	77.78	22	84.62	17	89.47	12	85.71
Aztreonam	67	44.67	25	45.45	16	44.44	11	42.31	5	26.32	10	71.43
Meropenem	132	88.00	43	78.18	30	83.33	26	100.00	19	100.00	14	100.00
Cotrimoxazole	57	38.00	27	49.09	0	0.00	13	50.00	12	63.16	5	35.71

### 3.5 Antimicrobial susceptibility among different periods

**Figure 4:**
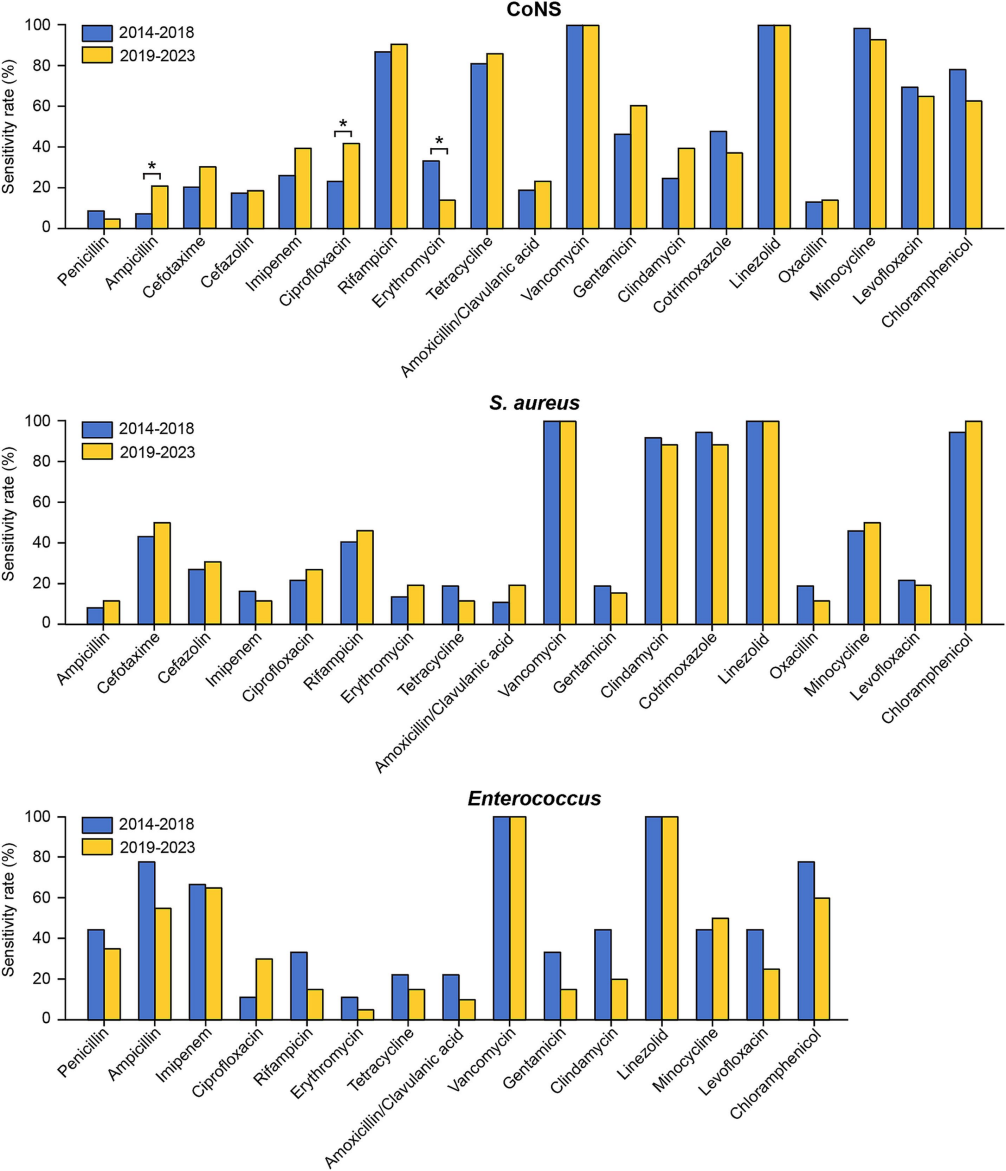
Comparison of the antimicrobial susceptibility of the predominant Gram-positive bacteria causing PCNSI in different periods. *p < 0.05. *Staphylococcus aureus* isolates exhibited complete (100%) resistance to penicillin, while *Enterococcus* isolates exhibited complete (100%) resistance to cefotaxime, cefazolin, cotrimoxazole, and oxacillin. These results were not presented in the figure.

**Figure 5:**
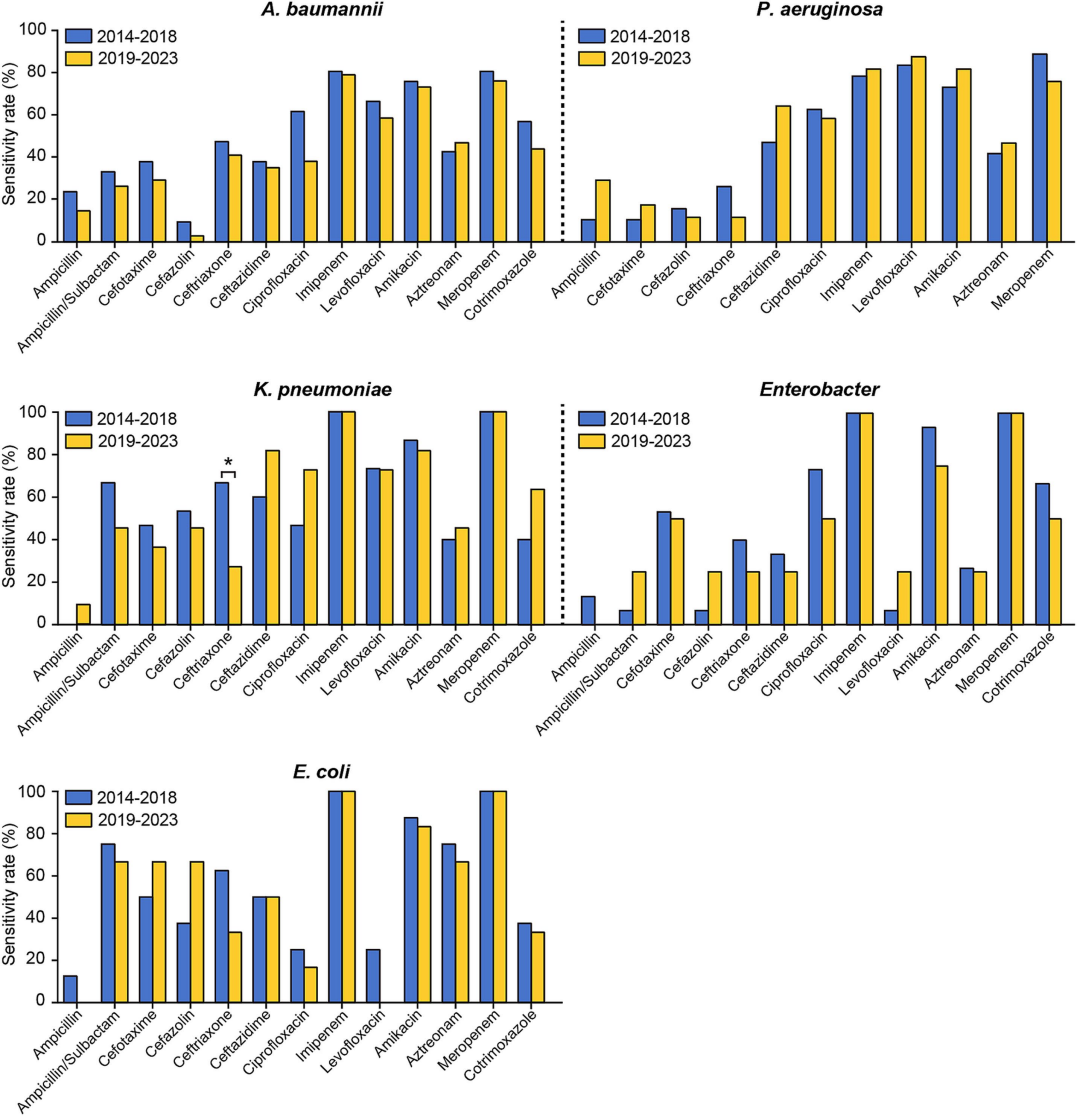
Comparison of the antimicrobial susceptibility of the predominant Gram-negative bacteria causing PCNSI in different periods. *p < 0.05. *Pseudomonas aeruginosa* isolates exhibited complete (100%) resistance to ampicillin/sulbactam and cotrimoxazole. These results were not presented in the figure.

The susceptibility rates of pathogenic strains in the two periods to commonly used drugs from January 2014 to December 2023 were also compared. As shown in Figure 4 and Supplementary Table S3, the susceptibility of coagulase-negative staphylococci to erythromycin significantly decreased from the first period to the second period. Conversely, coagulase-negative staphylococci exhibited significant increases in susceptibility to both ciprofloxacin and ampicillin. Ceftriaxone and cefazolin, two of the most commonly utilized cephalosporins in neurosurgical procedures, displayed distinct resistance trends in our study. The sensitivity of *Klebsiella pneumoniae* to ceftriaxone decreased markedly, from 66.67% in the first stage to 27.27% in the second stage (Figure 5 and Supplementary Table S4). Conversely, the resistance rates to cefazolin remained consistent across the different periods, demonstrating sustained efficacy against *Klebsiella pneumoniae* and *Escherichia coli* but persistently low sensitivity against *Enterococcus* (Figures 4, 5, and Supplementary Tables S3, S4).

## 4 Discussion

Despite dramatic advancements in neurosurgical techniques and postoperative management, the incidence of PCNSI has not demonstrated a proportional decline in recent decades (13). Evidence has indicated that the actual incidence of PCNSIs may greatly exceed currently reported rates (14). Therefore, for patients suspected of having postoperative infection, early diagnosis, the selection of appropriate antibiotics according to epidemiologic trends, susceptibility testing, and correction of targeted antimicrobial therapy are particularly critical for maximizing the potential for survival (Supplementary Figure S2).

A multitude of risk factors, such as shunt type, operative duration, surgeon experience, and hydrocephalus etiology, have been recognized as accountable for PCNSI. Although previous studies have consistently identified patient age and specific procedure types as potential contributors (15–18), our comparative analysis offers a distinct perspective. Contrary to the conventional understanding that CSF drainage is associated with a high risk of infection, our findings revealed that surgery types were not significantly correlated with PCNSI following neurosurgical procedures. Moreover, despite our assumption that older adults individuals might be more prone to PCNSI due to their lower immunity, our data indicated that age was not a statistically significant risk factor for PCNSI. This disparity may be attributed to the standardized infection control protocols implemented across all procedures in our institution or the relatively homogeneous risk profile of the patient population.

Over 100 pathogens have been identified as being associated with PCNSI after neurosurgical procedures, and the epidemiology of PCNSI exhibits temporal variations and varying geographic distributions (19). Therefore, a comprehensive definition of the differences in pathogen distribution could provide critical insights for the selection of appropriate empirical treatments (20). Among the 385 pathogens detected in the present study, the percentages of Gram-positive bacteria, Gram-negative bacteria, and fungi identified in our study were 56.10, 41.30, and 2.60%, respectively (Table 1 and Figure 1). This distribution pattern shows a high degree of consistency with the epidemiological data reported from three major Chinese medical centers located in the northern, central, and southern regions of China (20–22). Several recent studies from Europe and North America revealed similar patterns, with Gram-positive bacteria being predominant (23, 24). Overall, our study not only revealed the regional pathogen distribution profile but also reflected a similar global epidemiological trend, thereby providing valuable insights for the development of regional diagnostic strategies for PCNSI.

Among the 396 patients diagnosed with PCNSI in our study, only 6 (1.52%) had polymicrobial infections. This prevalence rate is notably lower than the 6.8–10% reported in previous studies (9, 25–27). One possible explanation for this discrepancy is the limited sensitivity of the conventional culture methods used in our study for detecting polymicrobial infections. These limitations might have led to some cases of polymicrobial infections remaining undetected, thus potentially lowering the recorded incidence. Moreover, our exclusion criteria exclude patients with severe organ dysfunction or immunocompromised status, who typically have a relatively high probability of developing polymicrobial infections. By excluding them from the study cohort, the overall incidence of polymicrobial infections was inevitably reduced. These factors may collectively have contributed to the lower detection rate of polymicrobial infections in our study.

With respect to the distribution of pathogenic bacteria in different periods, we found that coagulase-negative staphylococci were still the predominant pathogens in such patients, accounting for 32.70 and 24.71% of the total pathogens in the first and second periods, respectively. Coagulase-negative staphylococci are a part of the normal skin flora and frequently colonize intracranial devices (28, 29). Therefore, intracranial devices are considered high risk factors for the development of infections, leading to a high frequency of PCNSI caused by coagulase-negative staphylococci (13, 30, 31). Furthermore, an obvious increase in *Acinetobacter baumannii* was observed, which is consistent with trends reported in recent studies indicating increasing numbers of PCNSI cases caused by Gram-negative bacteria (20, 32). One possible explanation for this elevated prevalence is the escalating selection pressure imposed by the widespread use of carbapenem for Gram-negative prophylaxis in neurosurgery. Moreover, the biofilm-forming ability of *Acinetobacter baumannii* may lead to cumulative contamination of medical devices or other environments, thus increasing the risk of continuous dissemination and infection. Our data indicate that the management of coagulase-negative staphylococci and *Acinetobacter baumannii* is a major clinical challenge, and clinical surveillance of these pathogens needs to be imposed in the future.

The inappropriate use of antibiotics has led to increased antibiotic resistance (33), posing significant challenges to the use of existing therapeutic options for PCNSI after neurosurgical procedures. Understanding the resistance profile of the pathogenic bacteria causing PCNSI, especially in the last decade, may guide clinicians in implementing successful therapeutic strategies. According to the results of antibiotic sensitivity testing in this study, the most effective antibiotics against Gram-positive bacteria were vancomycin and linezolid (Table 3 and Figure 3). Vancomycin is used as the final line of treatment for severe infections caused by Gram-positive bacteria (34). Previous research has reported the emergence of vancomycin-resistant *Enterococcus* (21); however, no vancomycin-resistant isolates were detected in our study. For the Gram-negative bacteria, imipenem and meropenem were the most effective antibiotics (Table 4 and Figure 3), which was similar to the results of previously published studies (9, 20). Despite imipenem exhibiting potent activity against Gram-negative bacteria, its use is typically associated with a high risk of neurotoxic side effects, especially in neurosurgical patients (35, 36). Considering these pharmacodynamic properties and safety profiles, the combination of vancomycin and meropenem may be the predominant clinical regimen for treating PCNSI. Our proposed therapeutic protocol is consistent with the clinical practice guidelines of the Infectious Diseases Society of America (IDSA), which recommend vancomycin combined with cefepime, ceftazidime, or meropenem as appropriate empiric antimicrobial options for PCNSI (37).

Notably, our study revealed generally increased resistance to common antibiotics in several Gram-negative species, especially *Klebsiella pneumoniae* and *Acinetobacter baumannii* (Figure 5 and Supplementary Table S4). These observed trends are likely driven by complex interactions between microbial and therapeutic factors. *Acinetobacter baumannii* utilizes multiple resistance mechanisms, such as the production of β-lactamase, the activation of efflux pumps, and modifications of target sites (38, 39). These mechanisms have gradually accumulated over time because of the widespread use of broad-spectrum antibiotics. Moreover, previous studies reported that *Klebsiella pneumoniae* acquired two resistance plasmids harboring multiple antimicrobial resistance genes during evolution (40, 41). These adaptive features have contributed to a persistent increase in antibiotic resistance, conferring a considerable survival advantage to these pathogens throughout the evolutionary process. The prudent use of antibiotics, which requires considering both the risk to individual patients if not covered appropriately for a certain pathogen and the risks associated with an increase in resistant strains owing to overuse of antibiotics, will be critical in the future.

Our study cohort included 396 PCNSI cases and represents one of the larger datasets in this field, exceeding the median sample size (*n* = 155) reported in comparable studies published over the past 5 years. This substantial sample size provides sufficient statistical power for analyzing resistance trends among the predominant pathogens in the patient population. Notably, our microbial culture effectively identified several fewer common pathogens, including *C. neoformans* (1.04%), *C. albicans* (0.78%), *A. fumigatus* (0.52%), and *C. tropicalis* (0.26%) (Table 1 and Figure 1B). The detection of these pathogens, despite their relatively low prevalence, underscores the comprehensiveness of our diagnostic approach and surveillance system. However, we acknowledge that the limited quantities of these rare pathogens prevented us from conducting reliable antibiotic susceptibility testing or in-depth comparative analyses. To address this challenge, future studies should expand the surveillance scope and initiate multicenter collaborations to accumulate larger datasets for these uncommon pathogens.

The emergence of antibiotic resistance represents a complex biological survival response of bacteria. The fundamental mechanisms by which bacteria acquire or develop resistance mainly include the following: (1) the production of inactivating or modifying enzymes, such as β-lactamases and aminoglycoside-modifying enzymes; (2) the reduction of intracellular antibiotic concentrations through either poor penetration into bacteria or antibiotic efflux; (3) the modification of antibiotic target sites, such as ribosomal target sites and penicillin-binding proteins, via genetic mutation or posttranslational modification; and (4) the acquisition of resistance genes through horizontal gene transfer within and between species (42–45). To effectively address the escalating antimicrobial resistance crisis, healthcare institutions need to implement comprehensive, institutionally coordinated countermeasures to curtail the emergence and spread of drug-resistant pathogens. The rational use of antibiotics is an effective approach to reduce selection pressure. Formalized and practical guidelines for appropriate antibiotic prescription should be developed and implemented to prevent the overuse and misuse of antibiotics. Moreover, an appropriate cycling strategy, particularly periodic antibiotic monitoring and supervision on the basis of antibiotic heterogeneity, can mitigate antibiotic resistance. Additionally, a hierarchical management system should be established to optimize antimicrobial therapy and reduce or stabilize antimicrobial resistance.

In addition to antibiotic selection strategies, healthcare institutions should adopt multimodal control strategies to maintain stable antimicrobial resistance rates. First, hospital infection control should be intensified to curtail the dissemination of antibiotic-resistant infections, which encompasses hand hygiene management, environmental cleaning and disinfection, and isolation measures. Second, healthcare institutions should enhance the surveillance of drug-resistant bacteria, establish a surveillance system with information-sharing mechanisms, regularly report data on drug resistance patterns, and conduct targeted active screening. Moreover, healthcare institutions should improve training programs for medical staff, covering antimicrobial stewardship and infection prevention and control practices. These infection control measures can effectively reduce the risk of nosocomial transmission of resistant pathogens, thereby laying a foundation for future antimicrobial stewardship programs.

Despite its relative rarity, PCNSI is a serious complication in patients following neurosurgery, resulting in poor prognosis and high mortality risk (46). Therefore, the prevention and treatment of PCNSI remain great clinical challenges, with limited availability of effective antimicrobial agents. In this study, we conducted a retrospective epidemiological analysis of PCNSI at a tertiary hospital in North China. Our research contributes to a more comprehensive understanding of the distribution of pathogenic bacteria involved in PCNSI and changes in resistance trends, which will help in the development of new strategies for the prevention and treatment of PCNSI.

## 5 Limitations

The present study had several limitations. First, although the present study analyzed a greater number of pathogens than some previous studies did, selection bias was still present, and data from a single institution may not be representative of the entire population of China. Second, the conventional culture methods used in our study are likely inadequate for certain fastidious pathogens, especially fungi. Specifically, only 10 strains of fungi were identified in our study, and nearly 20 clinically confirmed patients with PCNSI failed to yield any identifiable pathogens. The restricted number of fungi made it impossible to perform reliable antibiotic susceptibility testing or conduct in-depth comparative analyses. Moreover, the exclusion of CSF-negative cultures due to technical limitations may have undermined the comprehensive characterization of the pathogen spectrum. Third, only 1.52% of patients were diagnosed with polymicrobial infections in our study. The limited number of pathogens isolated from these CSF samples restricts our understanding of the distinctive clinical and microbiological features of these complex infections. Future studies should expand the surveillance scope, optimize the culture and identification methods, and systematically increase the sample size to improve diagnostic accuracy and conduct more comprehensive analyses.

## 6 Conclusion

PCNSI is a serious complication associated with high mortality risk. In recent years, the increasing prevalence of antibiotic-resistant pathogens has complicated the existing empirical antibiotic therapy for PCNSI after neurosurgical procedures. Antimicrobial therapy should be modified according to the distribution of causative pathogens and the prevalence of antibiotic-resistant strains. In this study, we isolated and identified the pathogens causing PCNSI and assessed their antibiotic resistance during two periods from 2014 to 2023. Although Gram-positive bacteria, particularly coagulase-negative staphylococci, remain the predominant pathogens responsible for PCNSI following neurosurgery, the increasing proportion of infections caused by *Acinetobacter baumannii* and *Enterococcus* demands close monitoring and timely intervention in the future. Furthermore, our study revealed a concerning trend toward increased resistance to common antibiotics among several Gram-negative species, especially *Klebsiella pneumoniae* and *Acinetobacter baumannii*. In particular, *Acinetobacter baumannii*, which continues to exhibit increased resistance to common antibiotics and for which reliable therapeutic options are unavailable, poses a significant and persistent clinical challenge in clinical management. Our findings provide valuable insights for clinicians in optimizing empirical treatment strategies and highlight the necessity for continuous surveillance to address emerging resistance patterns in PCNSI pathogens.

## Data Availability

The original contributions presented in the study are included in the article/Supplementary material, further inquiries can be directed to the corresponding authors.
