# Supplementary material for: Prevalence and antibiotic resistance of pathogens isolated from neurosurgical patients with postoperative central nervous system infections in a tertiary hospital in North China
Source: Front Public Health. 2025 Jun 10;13:1601107. doi: 10.3389/fpubh.2025.1601107 (PMC12185526; doi:10.3389/fpubh.2025.1601107)
Supplement: Supplementary file 1 [file Data_Sheet_1.docx]

Supplementary Material

# Supplementary Figures and Tables

## Supplementary Figures


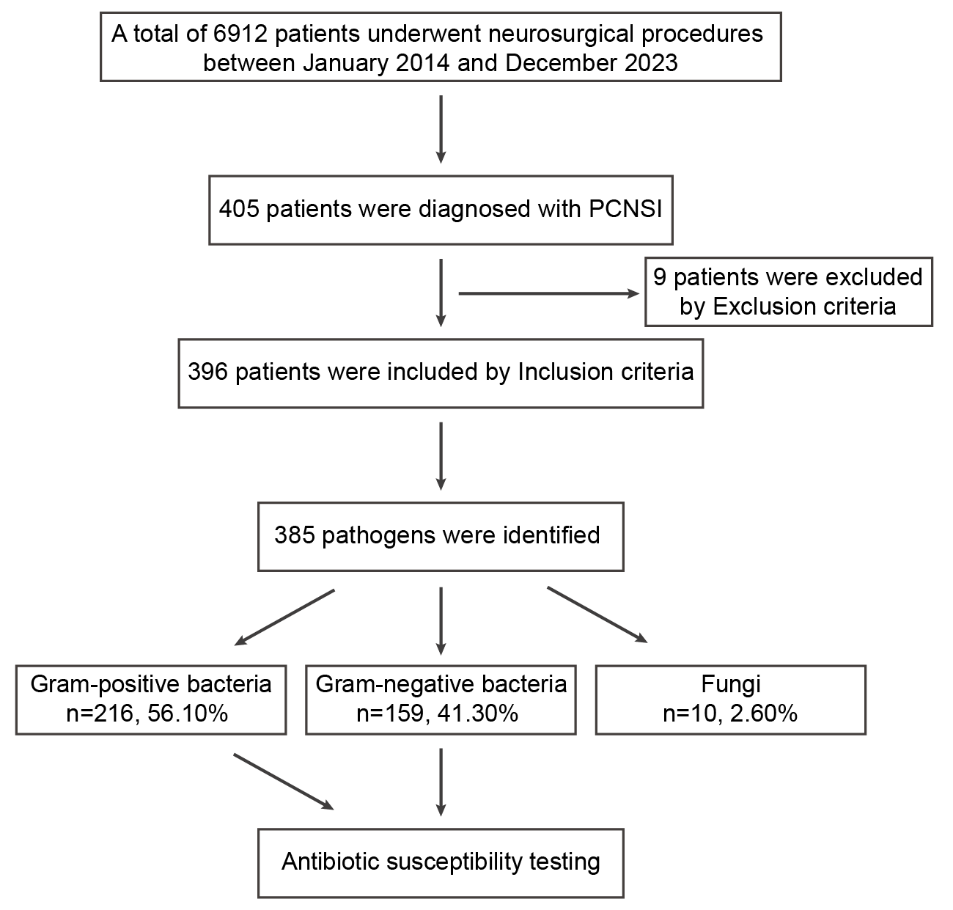


**Supplementary Figure 1.** Flowchart of the research process in our study.


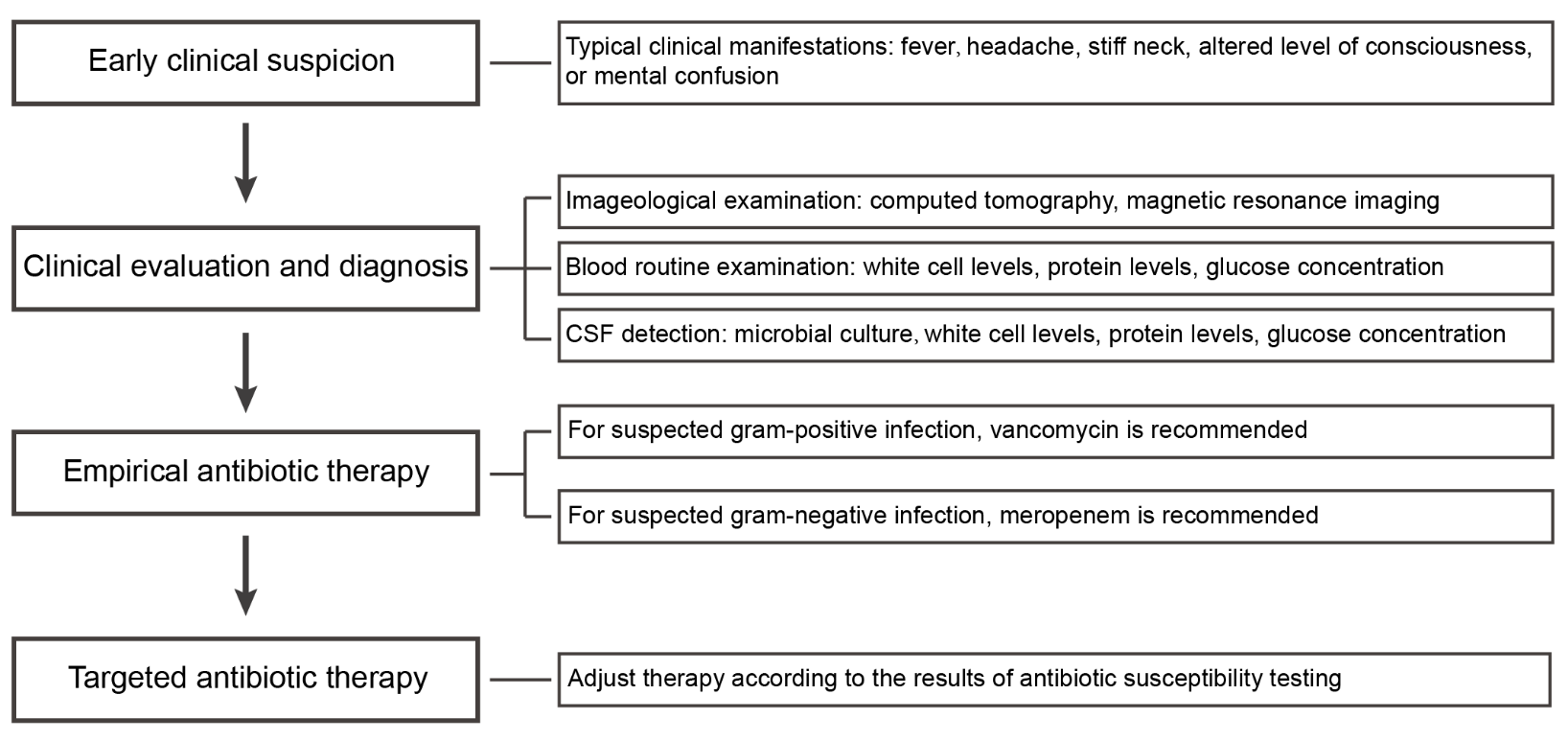


**Supplementary Figure 2.** Flowchart of the management protocol for patients suspected of having postoperative infection.

## Supplementary Tables

**Supplementary Table 1. Demographic and operational characteristics of patients with PCNSI from 2014–2023.**

| **Subgroup** | **Number (n=396)** | |
| --- | --- | --- |
|  | **2014–2019 (n=218)** | **2019–2023 (n=178)** |
| Age | | |
| Median (range) | 47 (19-68) | 44 (20-76) |
| Sex (n, %) | | |
| Male | 97, 44.50% | 85, 47.75% |
| Female | 121, 55.50% | 93, 52.25% |
| Procedure (n, %) | | |
| Cranial | 196, 89.91% | 162, 91.01% |
| Spinal | 22, 10.09% | 16, 8.99% |
| Operation time (n, %) | | |
| ≤4 h | 129, 59.17% | 91, 51.12% |
| >4 h | 89, 40.83% | 87, 48.88% |

**Supplementary Table 2. Pathogens isolated from 6 patients with polymicrobial infections from 2014–2023.**

| **Patient** | **Pathogens** |
| --- | --- |
| 1 | *Escherichia coli*; *Enterobacter*; *Pseudomonas aeruginosa* |
| 2 | *Enterobacter*; *Escherichia coli* |
| 3 | *Acinetobacter baumannii*; coagulase-negative staphylococcus |
| 4 | *Pseudomonas aeruginosa*; *Staphylococcus aureus* |
| 5 | *Klebsiella pneumoniae*; *Pseudomonas aeruginosa* |
| 6 | *Acinetobacter baumannii*; *Enterobacter* |
